# Supplementary material for: Causal Relationship between Aging and Anorexia Nervosa: A White-Matter-Microstructure-Mediated Mendelian Randomization Analysis
Source: Biomedicines. 2024 Aug 16;12(8):1874. doi: 10.3390/biomedicines12081874 (PMC11351342; doi:10.3390/biomedicines12081874)
Supplement: Supplementary file 1 [file biomedicines-12-01874-s001.zip › Supplementary Figure/Figure S4/Singe SNP plot of MR analysis for TL to ubm-b-1976.pdf]

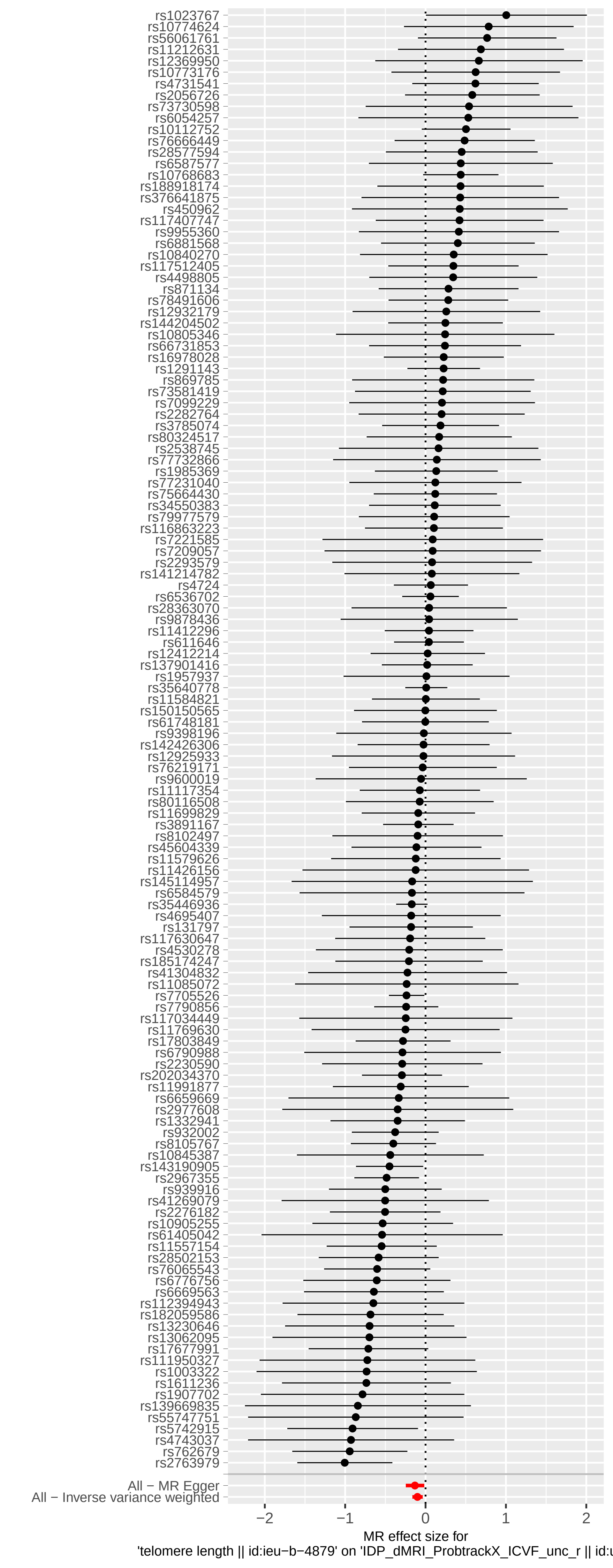
rs1023767
rs10774624
rs56061761
rs11212631
rs12369950
rs10773176
rs4731541
rs2056726
rs73730598
rs6054257
rs10112752
rs76666449
rs28577594
rs6587577
rs10768683
rs188918174
rs376641875
rs450962
rs117407747
rs9955360
rs6881568
rs10840270
rs117512405
rs4498805
rs871134
rs78491606
rs12932179
rs144204502
rs10805346
rs66731853
rs16978028
rs1291143
rs869785
rs73581419
rs7099229
rs2282764
rs3785074
rs80324517
rs2538745
rs77732866
rs1985369
rs77231040
rs75664430
rs34550383
rs79977579
rs116863223
rs7221585
rs7209057
rs2293579
rs141214782
rs4724
rs6536702
rs28363070
rs9878436
rs11412296
rs611646
rs12412214
rs137901416
rs1957937
rs35640778
rs11584821
rs150150565
rs61748181
rs9398196
rs142426306
rs12925933
rs76219171
rs9600019
rs11117354
rs80116508
rs11699829
rs3891167
rs8102497
rs45604339
rs11579626
rs11426156
rs145114957
rs6584579
rs35446936
rs4695407
rs131797
rs117630647
rs4530278
rs185174247
rs41304832
rs11085072
rs7705526
rs7790856
rs117034449
rs11769630
rs17803849
rs6790988
rs2230590
rs202034370
rs11991877
rs6659669
rs2977608
rs1332941
rs932002
rs8105767
rs10845387
rs143190905
rs2967355
rs939916
rs41269079
rs2276182
rs10905255
rs61405042
rs11557154
rs28502153
rs76065543
rs6776756
rs6669563
rs112394943
rs182059586
rs13230646
rs13062095
rs17677991
rs111950327
rs1003322
rs1611236
rs1907702
rs139669835
rs55747751
rs5742915
rs4743037
rs762679
rs2763979
All − MR Egger
All − Inverse variance weighted
−2
−1
0
1
2
MR effect size for
'telomere length || id:ieu−b−4879' on 'IDP_dMRI_ProbtrackX_ICVF_unc_r || id:u
